# Identification and analysis of exosome-associated signatures in pediatric sepsis by integrated bioinformatics analysis and machine learning

**DOI:** 10.7717/peerj.20555

**Published:** 2026-01-08

**Authors:** Junming Huang, Lichuan Lai, Jinji Chen, Xiaotao Su

**Affiliations:** 1 Department of Neurology, First Affiliated Hospital of Guangxi Medical University, Nanning, China; 2 Department of Laboratory, The People’s Hospital of Guangxi Zhuang Autonomous Region, Nanning, China; 3 Deparment of Urology, First Affiliated Hospital of Guangxi Medical University, Nanning, China

**Keywords:** Pediatric sepsis, Exosome-related genes, Machine learning, WGCNA, Immune microenvironment

## Abstract

**Background:**

Pediatric sepsis (PS) is a critical condition characterized by life-threatening organ dysfunction and immune dysregulation, including exosome-mediated immune modulation, often linked to infections. Investigating the role of exosome-related genes (ERGs) in the pathogenesis of PS is essential for identifying significant diagnostic and therapeutic targets.

**Methods:**

Four datasets, namely GSE66099 (training set) and GSE13904, GSE26378, and GSE26440 (validation sets), were retrieved from the Gene Expression Omnibus (GEO). The differential expression of 56 ERGs was analyzed, followed by consensus clustering to identify distinct exosome-related patterns in PS. Weighted gene co-expression network analysis (WGCNA) was utilized to identify PS-related genes (SRGs). Additionally, the immune microenvironment was assessed, and diagnostic models were developed employing specific machine learning algorithms.

**Results:**

The differential expression analysis identified 21 ERGs that exhibited significant alterations in PS. Consensus clustering revealed two distinct subtypes of PS based on the expression pattern of ERGs. WGCNA identified several hub genes involved in exosome function and PS, with immune-related pathways, including phagocytosis and NF-κB signaling, showing significant enrichment. These genes were leveraged to construct machine learning models, which demonstrated a high diagnostic accuracy, with an area under the curve (AUC) > 0.995. The analysis identified *CD177*, *GYG1*, *IRAK3*, *MCEMP1*, and *TLR5* as key biomarkers. Furthermore, external validation confirmed the superior performance of the constructed model.

**Conclusion:**

This study elucidated the role of ERGs in PS, and highlights the significance of immune dysregulation in the pathogenesis of the disease. The developed diagnostic models represent promising tools for the early detection and prognosis prognostic of PS.

## Introduction

Pediatric sepsis (PS) is a highly complex and life-threatening syndrome that affects millions worldwide each year (Weiss & Fitzgerald, 2024). The incidence and mortality rates are highest among the elderly, neonates, and young children (Watson et al., 2024). According to the 2005 International Pediatric Sepsis Consensus Conference, PS is defined as suspected or proven infection accompanied by systemic inflammatory response syndrome; severe sepsis is sepsis with cardiovascular or respiratory dysfunction or dysfunction of two or more other organ systems; and septic shock is sepsis with hypotension, requirement for vasoactive agents, or impaired perfusion despite at least 40 mL/kg fluid resuscitation (Schlapbach et al., 2024). In pediatric populations, the incidence of PS gradually decreases from birth to 19 years of age, reaching 89 cases per 100,000 individuals. However, the burden is considerably higher in neonates, among whom group B streptococcus represents a major causative pathogen (Manuel et al., 2025). For five years of age, sepsis and infections account for 6.3 deaths per 1,000 live births. The estimated case-fatality rate of PS upon diagnosis is approximately 25% (Weiss et al., 2015). Despite significant advancements in scientific understanding, there remains a pressing need to enhance our knowledge of the pathophysiology of PS, identify key risk factors, and refine management strategies. The development of innovative therapeutic approaches is essential for improving patient outcomes.

Studies have demonstrated that patients with severe infections often experience a hyperinflammatory response, which contributes to the development of multiple organ dysfunction (Lelubre & Vincent, 2018). Based on this background, anti-inflammatory therapies are frequently employed to manage PS patients, and a variety of strategies aimed at modulating inflammation are being increasingly developed (Zhou et al., 2021; Liu et al., 2022).

Immunosuppression in these patients is influenced by several factors, including individual frailty, underlying health condition, and inadequate immune responses to infections, all of which elevate the risk of mortality associated with acute inflammation, PS, and infections (Rice et al., 2018; Huang et al., 2025). In this context, maintaining a balanced immune response is critical for effective microbial eradication and the prompt resolution of PS (Shen et al., 2024). In the initial stages of PS, innate immune cells such as macrophages and dendritic cells recognize invading pathogens and rapidly produce pro-inflammatory cytokines, among which interleukin-1 (IL-1) plays a central role. IL-1 is generated as an inactive precursor (pro-IL-1β) and cleaved into its active form *via* inflammasome-dependent caspase-1 activation (Caceres et al., 2024). Once secreted, IL-1 binds to the IL-1 receptor on target cells, activating downstream signaling pathways including NF-κB and mitogen-activated protein kinase (MAPK), which induce the expression of additional pro-inflammatory mediators, chemokines, and adhesion molecules (Lima, 2023). This cascade results in recruitment of neutrophils and monocytes to infection sites, increased vascular permeability to facilitate immune cell trafficking, and induction of fever through hypothalamic signaling, collectively enhancing pathogen clearance (Xie et al., 2024). However, if the inflammatory response becomes excessive or prolonged, counter-regulatory anti-inflammatory pathways (*e.g.*, IL-10, TGF-β) may be overactivated, leading to immune cell dysfunction, impaired antigen presentation, and T-cell suppression (Carlini et al., 2023). This dysregulation ultimately causes immunosuppression, increasing susceptibility to secondary infections and contributing to organ dysfunction and poor clinical outcomes (Hotchkiss, Monneret & Payen, 2013; Nolt et al., 2018; Green et al., 2023). This intricate process affects the immune status of patients with PS, yet the profile of immune regulation in this condition remains poorly understood (Piliponsky et al., 2019).

To further investigate the intricate mechanisms of PS, recent advancements have underscored the importance of exosomes, a vital type of extracellular vesicle that facilitates cell-to-cell communication across various physiological processes (Hwang, Shimizu & Lee, 2022). Exosomes, typically ranging from 30 to 150 nm in diameter, transport a range of bioactive substances, including proteins and lipids, which collectively contribute to their biological functions and high biocompatibility. Recently, substantial progress has been made in exosome research, particularly in relation to cardiovascular diseases (Li et al., 2019), neurological disorders (Danielski et al., 2020), stem cell biology (Zhao et al., 2019), and notably, sepsis (Jiao et al., 2023). However, the relationship between exosome-related genes (ERGs) and PS remains unexplored. Consequently, examining the molecular subtypes and genomic diversity of PS, particularly concerning exosomes and their key driver genes, is essential for enhancing our understanding of the critical pathogenic mechanisms that drive the development and progression of PS.

In our study, we hypothesize that ERGs play a pivotal role in modulating immune dysregulation and disease progression in PS. Therefore, by integrating bioinformatic analyses and machine learning techniques, we aim to systematically characterize the expression patterns of ERGs, identify potential molecular subtypes, and uncover key diagnostic biomarkers and therapeutic targets that may facilitate early diagnosis and personalized treatment of PS.

## Methods

### Data collection and dataset information

Four datasets were obtained from the Gene Expression Omnibus (GEO) database. GSE66099, containing the largest sample size, was used as the training cohort, whereas GSE13904, GSE26378, and GSE26440, with smaller but comparable sample sizes and consistent clinical phenotypes, served as independent validation cohorts. Detailed information about these datasets is provided in Table S1.

### Differential expression analysis of ERGs

An extensive literature review was conducted using “exosome” as the search term in the GeneCards database, applying a relevance score cutoff of 6.0. This review identified 56 ERGs. Differential expression analysis of these ERGs between PS and healthy samples was performed using the R package “limma”, with results were visualized through the “ggpubr” and “pheatmap” packages. To explore potential interactions among the differentially expressed ERGs, a correlation plot was generated using the “circlize” package.

### Identification of exosome-related patterns

Consensus clustering analysis was performed on the GSE66099 training dataset based on ERG expression profiles utilizing the “ConsensusClusterPlus” package, which identified the optimal number of clusters. Principal component analysis (PCA) was then applied to visualize the distribution of exosome-related patterns following clustering.

### Gene set variation analysis

Enrichment analysis was conducted using “c5.go.symbols” and “c2.cp.kegg.v7.2.symbols”, in R package “GSVA” (gene set variation analysis), which were downloaded from the Molecular Signatures Database. Statistically significant pathways were displayed on a bar plot.

### Weighted gene co-expression network analysis

The relationship between gene modules and PS disease traits was investigated using weighted gene co-expression network analysis (WGCNA), which identified key hub genes implicated involved in the pathogenesis of PS. Genes exhibiting the top 25% variance were selected for analysis. A similarity matrix was constructed based on Pearson’s correlation coefficients. Subsequently, a topological overlap matrix (TOM) was generated, and modules were identified utilizing a dynamic tree-cut algorithm. Hub genes were selected based on the thresholds of gene significance (GS) > 0.2 and module membership (MM) > 0.6.

### Immune microenvironment

To estimate the relative proportions of 22 immune cell types, the CIBERSORT algorithm was employed. This method adaptively selects genes from a reference signature matrix for deconvolution. The estimated immune cell compositions were compared across groups, and their correlations with expression levels of ERGs were assessed.

### Diagnostic model construction and validation

Key genes identified through WGCNA were further analyzed to construct a diagnostic model for PS using multiple machine learning algorithms. The “kernlab”, “randomForest”, and “xgboost” R packages were utilized to implement Support Vector Machines (SVM), Random Forest (RF), and eXtreme Gradient Boosting (XGB) algorithms, respectively. Model development and resampling were performed within the “caret” R package using a 5-fold repeated cross-validation strategy to ensure robust training and prevent overfitting. Samples were randomly partitioned into training (70%) and testing (30%) cohorts, and all classifiers were fitted using standardized training procedures. Model interpretability, including residual diagnostics and variable-importance profiling, was assessed using the “DALEX” package. Diagnostic performance was evaluated on the held-out testing cohort using receiver operating characteristic (ROC) analysis implemented in the “pROC” package, through which five key predictive signatures were identified. External validation was conducted using independent datasets to further verify model robustness. To ensure computational reproducibility, all analysis scripts, model parameters, and trained model objects have been deposited in a public GitHub repository (https://github.com/gxmuhjm/exosome_sepsis.git) and permanently archived in Zenodo (https://doi.org/10.5281/zenodo.17626895).

### Quantitative polymerase chain reaction (qPCR)

Whole blood samples were collected from ten healthy individuals and nine PS patients for qPCR validation. Total DNA was extracted using the FastPure Blood DNA Isolation Mini Kit V2 (Sangon, Shanghai, China). The DNA was subjected to PCR using the FastStart Essential DNA Green Master reagent (Roche, Shanghai, China) on the LightCycler^®^ 96 Instrument (Roche Diagnostics GmbH, Switzerland). In the PCR assessment, β-Actin served as the internal control. Primer sequences are provided in Table S2. The studies involving human participants were approved by the First Affiliated Hospital of Guangxi Medical University (2025-E0058) and were conducted following local legislation and institutional requirements. Participants provided written informed consent to participate in this study.

### Statistical analysis

Non-normally distributed data were compared using non-parametric Wilcoxon tests, while Student’s t-tests were employed for normally distributed data. Spearman correlation tests were conducted to explore associations among variables. *: *P* < 0.05, **: *P* < 0.01, and ***: *P* < 0.001.

## Results

### Differential expression patterns of ERGs in PS

**Figure 1:**
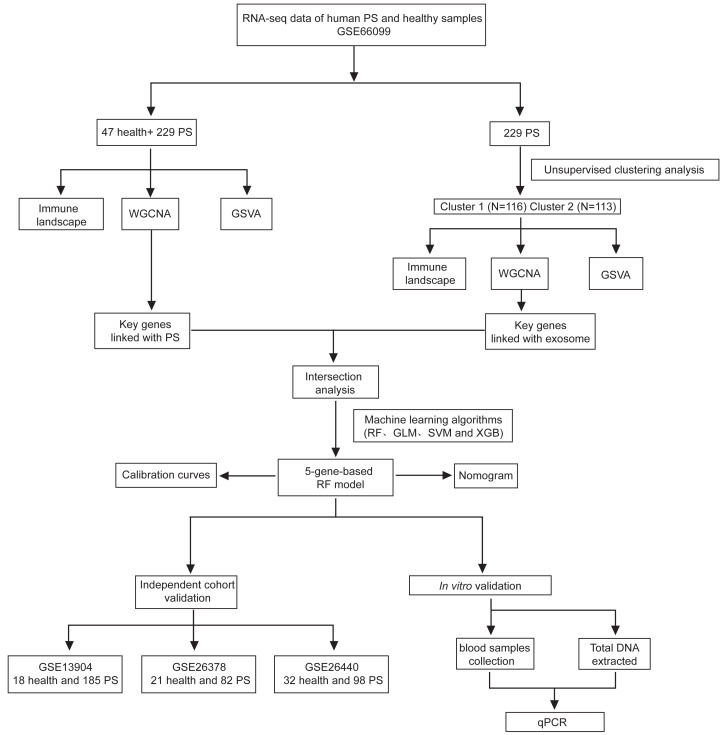
The flow chart of the study.

**Figure 2:**
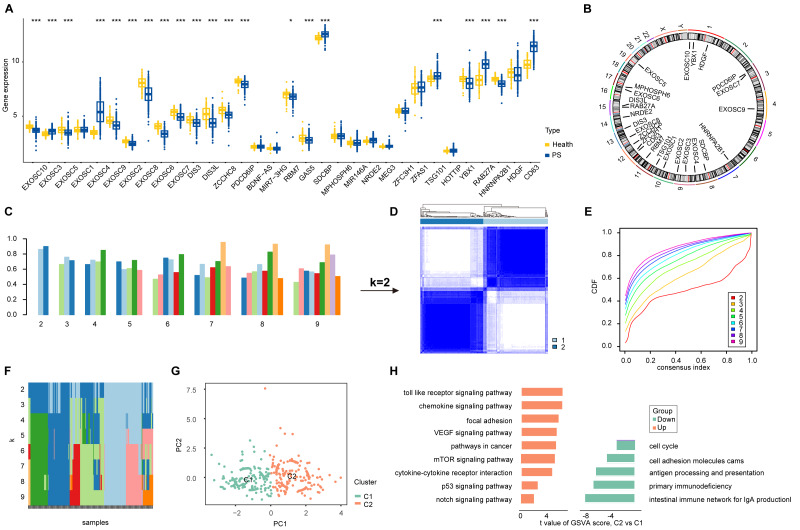
Illustration and cluster analysis of differentially expressed ERGs in PS samples. (A) Boxplot comparing the expression levels of 32 ERGs between healthy individuals and PS samples. (B) The genomic distribution of the 21 differentially expressed ERGs across chromosomes. (C) Consensus scores calculated for each k value. (D) Hierarchical clustering of PS samples into two distinct groups. (E) CDF for consensus clustering with k values ranging from 2 to 9. (F) Trace plot displaying the clustering patterns in all samples for k values between 2 and 9. (G) PCA showing the separation of the two clusters. (H) GSVA enrichment analysis among the ERGs in C1 and C2 clusters.

A schematic illustration of the study design is presented in Fig. 1. Initially, 56 ERGs were identified from public databases (details provided in Table S3). Subsequently, 21 of these ERGs were found to be differentially expressed in PS samples. Among these, EXOSC3, EXOSC4, SOCBP, TSG101, RAB27A and CD63 were significantly upregulated, while EXOSC10, EXOSC5, EXOSC9, EXOSC2, EXOSC8, EXOSC6, EXOSC7, DIS3, DIS3L, ZCCHC8, PDCD6IP, RBM7, GAS5, YBX1 and HNRNPA2B1 were downregulated in PS (Fig. 2A). The chromosomal locations of the differentially expressed ERGs are displayed in Fig. 2B. Subsequently, we characterized the immune microenvironment in PS, with the results visualized on a heatmap of immune cells (Fig. S1). Correlation analysis (Fig. S2A) revealed strong associations between ERGs and specific immune cell types. such as T cells and macrophages. These findings suggest a potential mechanistic interplay between exosome dynamics and immune regulation in PS. The various patterns of immune cell infiltration across different cohorts are illustrated in Fig. S2B. Collectively, these results underscore the critical role of ERGs in the pathological progression of PS and their regulatory networks within the immune microenvironment.

### Unsupervised clustering of differentially expressed ERGs

To investigate the heterogeneity in ERG expression, consensus clustering was conducted on 229 PS samples, yielding consistency scores exceeding 0.8 for both clusters when *k* = 2 (Fig. 2C). Consequently, we identified two distinct clusters of PS samples in subsequent analyses (Fig. 2D). The stability of these clusters was confirmed using consensus cumulative distribution function (CDF) curves (Fig. 2E) and trace plots (Fig. 2F). Cluster 1 (C1) comprised 116 samples, while Cluster 2 (C2) included 113 samples, with clear separation demonstrated through PCA (Fig. 2G). Differential expression analysis revealed significant differences in 21 of the 27 ERGs between C1 and C2 (Fig. S3). Additionally, GSVA indicated enrichment of infectious and intercellular communication-related pathways in C2, including toll-like receptor signaling, VEGF signaling, mTOR signaling, and p53 signaling, among others. In contrast, C1 was associated with immune-related pathways such as the intestinal immune network and antigen processing and presentation (Fig. 2H). Analysis of immune cell composition analysis demonstrated pronounced differences in 10 immune cell types between the clusters (Figs. S4A–S4B), providing insights into the distinct immunological landscapes within each cluster.

### WGCNA to identify hub PS-related genes (SRGs) and ERGs

**Figure 3:**
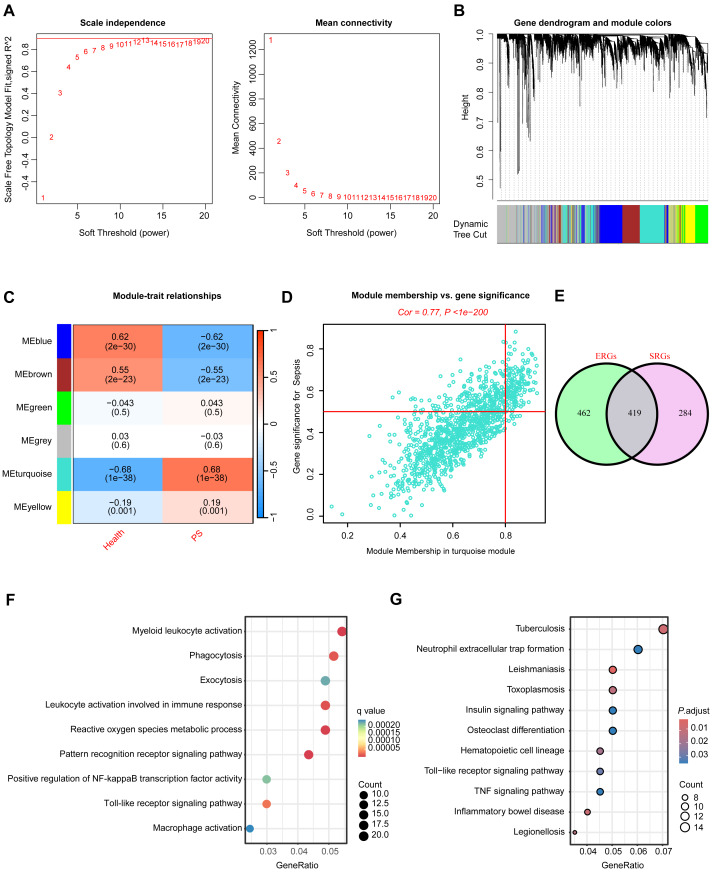
Identification of hub SRGs. (A) Soft-thresholding analysis for to facilitate network construction. (B) Clustering dendrogram illustrating gene hierarchical clustering. (C) Correlation heatmap depicting associations between SRGs modules and PS features. (D) Analysis of the correlation between turquoise module genes and PS. (E) Venn diagram illustrating the intersection of hub SRGs and ERGs. (F) GO and (G) KEGG enrichment analysis results for the shared genes.

To identify SRGs, the WGCNA algorithm was employed, followed by the construction of a scale-free network utilizing a soft threshold of 13, resulting in an R^2^ value of 0.98 (Fig. 3A). The evaluation of the sample clustering diagram revealed four distinct co-expression modules (Fig. 3B). Among the identified gene modules, the turquoise module exhibited a strong correlation with PS, with a coefficient of 0.68 and a *P*-value of e^−38^ (Fig. 3C). Furthermore, we observed a strong correlation between GS and MM within the turquoise module (Fig. 3D). This module contained 703 genes significantly associated with PS (Table S4). Among the exosome-related clusters, the WGCNA results indicated that the turquoise module was the most correlated with cluster classification (cor = 0.6, *P* = 2.9e^−87^). Within this module, 881 hub ERGs were strongly associated with PS (Figs. S5A–S5D, Table S5).

Intersection analysis of hub SRGs and ERGs revealed 419 shared genes (Fig. 3E). Gene Ontology (GO) enrichment analysis of these shared genes indicated significant enrichment in pathways critical for immune function and intercellular communication, including exocytosis, phagocytosis, pattern recognition receptor signaling, and toll-like receptor signaling (Fig. 3F). Kyoto Encyclopedia of Genes and Genomes (KEGG) pathway analysis identified significant enrichment in various pathways related to human diseases, genetic information processing, and cellular processes (Fig. 3G). In conclusion, these findings provide valuable insights into the molecular mechanisms underlying the interactions between PS and ERGs.

### Diagnostic model construction using machine learning

**Figure 4:**
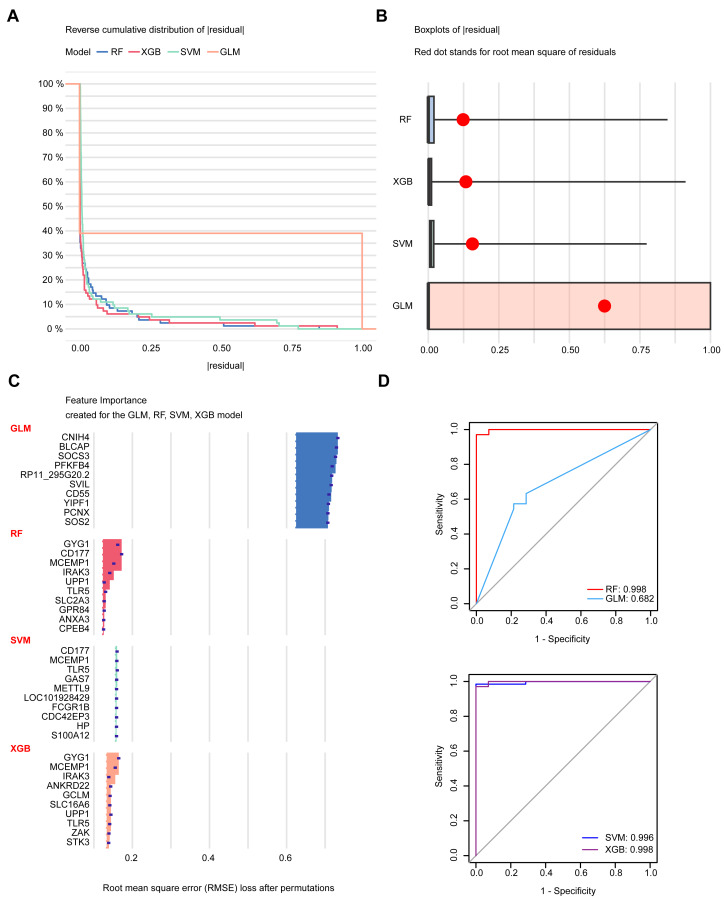
Construction of a diagnostic model. (A) Cumulative residual distribution plots for the four machine learning methods. (B) Boxplots displaying residuals of each model, with red dots indicating RMSE values. (C) Ranking of the top ten variables based on RMSE for each model. (D) ROC curve for the four machine learning methods.

To construct diagnostic models, RF, SVM, XGB, and Generalized Linear Model (GLM) were applied. Notably, the cumulative residual distribution plots (Fig. 4A) and boxplots of residuals (Fig. 4B) for the algorithms revealed that RF yielded the smallest residual values. Figure 4C presents the top ten variables for each model, ranked according to their root mean square error (RMSE). The diagnostic accuracy of these models was assessed using receiver operating characteristic (ROC) curves (Fig. 4D). The three models exhibited excellent discriminative performance, with an area under the curve (AUC) exceeding 0.995. Among them, RF proved to be the most reliable diagnostic model for PS, as evidenced by its predictive performance and stability. The top five most significant variables, CD177, GYG1, IRAK3, MCEMP1 and TLR5, were considered as key biomarkers for PS diagnosis.

Furthermore, a nomogram was constructed based on the identified hub genes (Fig. S6A). The calibration curve demonstrated excellent agreement between predicted and actual probabilities, indicating high predictive accuracy (Fig. S6B). Moreover, decision curve analysis (DCA) confirmed the clinical utility of this nomogram in facilitating clinical decision-making (Fig. S6C).

**Figure 5:**
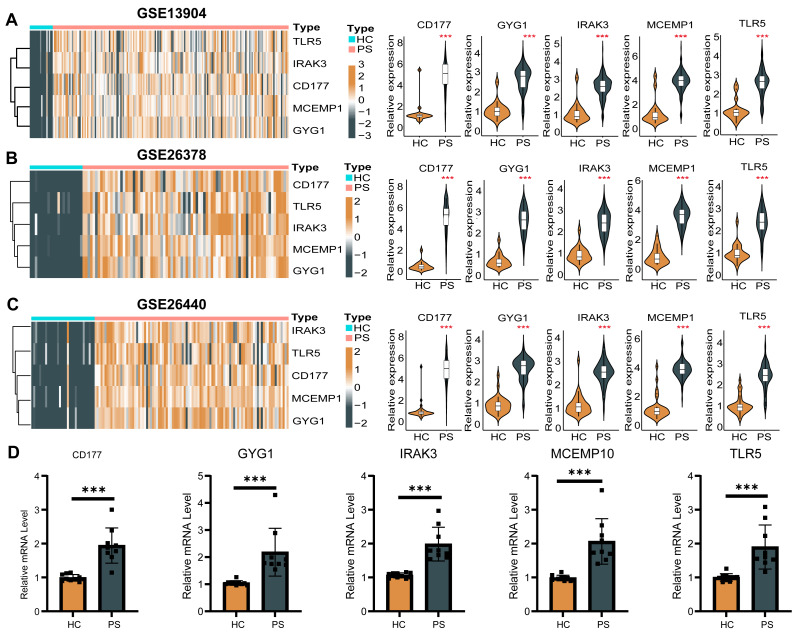
Validation of the expression of the five hub genes in diagnostic model through various independent validation dataset (A–C) and RT-qPCR (D). HC, healthy control.

Moreover, the performance of the five-gene diagnostic model was validated in external datasets (GSE13904, GSE26378, GSE26440), achieving high AUC values of 1.000 (Figs. 7A–S7C), with expression patterns consistent across these datasets (Figs. 5A–5C). The qPCR analysis was employed to validate the differential expression of the five model genes, which aligned with RNA-seq data (Fig. 5D). This further substantiates their diagnostic value in PS. In conclusion, we demonstrate that the application of machine learning techniques facilitated the establishment of a robust diagnostic model, which exhibited promising clinical predictive performance in PS.

## Discussion

In this study, the expression patterns of ERGs were explored to elucidate their potential roles in the pathogenesis of PS. A total of 56 ERGs were identified and subjected to gene expression profiling. Differential expression analysis revealed that 21 of the 56 ERGs were significantly altered in PS samples. Furthermore, consensus clustering and WGCNA were conducted to investigate the molecular mechanisms of these ERGs in PS. Predictive models were established using machine learning tools, and their performance was rigorously validated.

While significant advances have been made in the understanding and treatment of PS, including the implementation of the Sequential Organ Failure Assessment (SOFA) score, quick SOFA (qSOFA) score and pediatric SOFA (pSOFA) score (Tables S6A–S6C) to evaluate organ dysfunction (Shankar-Hari et al., 2016), current scoring systems do not entirely capture the variability in sub-score criteria (Sanchez-Pinto & Khemani, 2016). The diverse presentations of PS, along with variations in patient responses to treatment, continue to pose significant challenges in improving clinical outcomes. Ongoing research is crucial for deepening our understanding of the underlying mechanisms, identifying distinct PS subtypes, and developing targeted therapeutic strategies to enhance patient prognosis. Addressing these knowledge gaps and accurately classifying PS subtypes are essential priorities for advancing both research and clinical practice.

In recent years, significant progress has been achieved in diagnosing and treating conditions such as PS, particularly through the integration of machine learning techniques with various clinical phenotypes, including test results (Qin et al., 2022). This approach provides a valuable opportunity to explore the complexity of the disease and facilitate more accurate classifications, thereby advancing the field of precision medicine. Studies have shown that machine learning methods outperform traditional clinical practices in predicting severe forms of PS (Banerjee et al., 2021), identifying distinct PS subgroups (Qin et al., 2023), and supporting early personalized anti-inflammatory interventions with improved diagnostic accuracy and efficiency (Qin et al., 2022).

However, many prior studies have been limited by small sample sizes, inadequate validation sets, and reliance on a single algorithm, raising concerns regarding the reliability of existing diagnostic models and undermining their initial objectives. To address these challenges, our research aims to develop a more robust diagnostic model for PS by incorporating four distinct machine learning algorithms. Notably, the RF algorithm has demonstrated the most consistency and precision, exhibiting superior predictive power. In our study, five key genes were identified as central factors: *CD177*, *GYG1*, *IRAK3*, *MCEMP1*, and *TLR5*, which were subsequently examined for their roles in PS progression. To validate these findings, qPCR tests were conducted on peripheral blood samples from both PS patients and healthy controls. The five-gene diagnostic model exhibited exceptional performance and robustness when tested against three independent validation datasets. These results not only highlight the clinical potential of the model but also emphasize the critical role of these hub genes in the pathogenesis of PS.

In recent years, research has highlighted the critical roles of exosomes in PS pathophysiology (Yang et al., 2022). Exosomes, which facilitate cell-to-cell communication, are increasingly recognized for their involvement in immune regulation and inflammation (Zeng et al., 2022; Zuo et al., 2022). Specifically, they modulate PS by transmitting intracellular signaling molecules and actively participating in immune responses (Kalluri & LeBleu, 2020). Notably, exosomes carry bioactive molecules, such as cytokines and proteins, which influence the progression of PS by regulating immune cell functions and interactions. A previous study demonstrated that lipopolysaccharide (LPS) injection triggered endotoxemia resembling PS, resulting in a significant increase in exosome levels in the serum of mice (Gao et al., 2019). Similar findings were observed *in vitro*, where LPS treatment enhanced exosome production in cells (Ti et al., 2015). In a PS model induced by cecal ligation and puncture (CLP), neutrophil-derived extracellular vesicles were markedly elevated in the blood of the mice (Herrmann et al., 2015). Furthermore, the administration of GW4869, an inhibitor of exosome production, prolonged the survival of mice subjected to either LPS injection or CLP. These results indicate that exosomes are involved in the development of PS (Essandoh et al., 2015). In addition, a recent systematic review reported that transcript host-RNA signatures—distinct from exosomal RNA and derived from the transcriptional biosignatures of host leukocytes—represent a novel tool to differentiate viral from bacterial infections, providing new insights into the molecular mechanisms of PS (Buonsenso, Sodero & Valentini, 2022). Therefore, our findings suggest potential targets for early diagnosis and therapeutic interventions in PS.

Importantly, these findings also carry notable clinical implications. Although precision medicine approaches, such as transcriptomic profiling or multi-omics analyses, hold promise for optimizing the management of PS, their high costs currently limit widespread implementation. Therefore, more affordable and readily available surrogate biomarkers remain highly relevant in clinical practice. Among these, procalcitonin has been widely used to support the diagnosis and risk stratification of sepsis (Wei et al., 2025); however, its sensitivity and specificity are limited, as elevated levels may also occur in severe viral infections. Presepsin, a novel biomarker reflecting activation of the innate immune response, has demonstrated encouraging diagnostic and prognostic value in PS (Hincu et al., 2025), although it is not yet routinely available in all clinical centers. Furthermore, recent studies have highlighted the potential of exosomes and ERGs in clinical diagnosis and therapeutic intervention (Lee et al., 2025; Wu et al., 2025). Future strategies integrating these cost-effective biomarkers, ERGs, and clinical scoring systems such as pSOFA may facilitate early identification, risk stratification, and personalized management of PS while maintaining practicality and cost-effectiveness.

The primary objective of this study was to investigate the relationship between PS and ERGs. Neutrophils are crucial effector cells in innate immunity, responsible for pathogen phagocytosis, cytokine production, and antimicrobial activity (Liew & Kubes, 2019). However, in PS, neutrophil function is often suppressed, characterized by decreased capacity to clear pathogens, which leads to tissue damage and uncontrolled inflammation (Venet & Monneret, 2018). This immune dysregulation is associated with abnormal intracellular signaling, a process in which exosomes are pivotal (Jiao et al., 2023). Our study revealed that the expression of multiple ERGs was altered in PS, disrupting immune cell interactions and exacerbating immune dysregulation. This indicates that exosomes represent promising targets for the development of therapeutic strategies aimed at restoring immune balance and improving outcomes in PS.

Although we have established diagnostic models for PS and validated their performance, further clinical validation is necessary, particularly in diverse patient populations and clinical settings. In addition, as information on the causative pathogens was not available in the current study, the potential influence of different infectious organisms on our results remains unclear and warrants further investigation. Moreover, the mechanisms of exosomes require validation through experimental models. Furthermore, *in vitro* and animal studies are needed to confirm our findings.

## Conclusion

In conclusion, this study highlights the critical role of ERGs in the pathogenesis of PS. Our findings indicate that exosome-mediated modulation of immune function exacerbates PS. The diagnostic models developed in this study, utilizing ERGs, are anticipated to enhance the accuracy of PS diagnosis and prognosis. Further research into the molecular mechanisms underlying these interactions is imperative for developing robust targeted therapies to enhance outcomes in PS.

##  Supplemental Information

10.7717/peerj.20555/supp-1 Supplemental Information 1 Detailed information of the datasets

10.7717/peerj.20555/supp-2 Supplemental Information 2 Primer sequences used in RT-qPCR

10.7717/peerj.20555/supp-3 Supplemental Information 3 List of 56 ERGs

10.7717/peerj.20555/supp-4 Supplemental Information 4 A total of 703 hub module genes in the turquoise module through WGCNA

10.7717/peerj.20555/supp-5 Supplemental Information 5 A total of 881 hub module genes in the turquoise module through WGCNA

10.7717/peerj.20555/supp-6 Supplemental Information 6 Clinical scoring systems for sepsis evaluation (A) The SOFA score for adults. (B) The qSOFA score for adult rapid screening. (C) The pSOFA score for pediatrics. References

10.7717/peerj.20555/supp-7 Supplemental Information 7 Proportional distribution of 22 immune cell types infiltrating the samples

10.7717/peerj.20555/supp-8 Supplemental Information 8 ERGs correlations and immune Infiltration in PS (A) Correlation analysis demonstrating the interrelationships among 22 differentially expressed ERGs. (B) Violin plot indicating the differential abundance of infiltrating immune cells between PS and healthy samples.

10.7717/peerj.20555/supp-9 Supplemental Information 9 Boxplot comparing the expression levels of 21 ERGs between C1 and C2

10.7717/peerj.20555/supp-10 Supplemental Information 10 CIBERSORT analysis of the infiltrating immune cells in consensus clusters (A) Proportion of 22 immune cell types infiltrating the clusters. (B) Violin plot demonstrating the differential immune cell abundance between C1 and C2 clusters.

10.7717/peerj.20555/supp-11 Supplemental Information 11 Identification of hub ERGs (A) Soft-thresholding procedure used for network construction. (B) Hierarchical clustering dendrogram of genes. (C) Correlation heatmap showing the correlation between ERGs modules and cluster features. (D) Association of turquoise module genes with exosome-related processes.

10.7717/peerj.20555/supp-12 Supplemental Information 12 Validation of the diagnostic model (A) Nomogram constructed using the 5 hub genes (CD177, GYG1, IRAK3, MCEMP1, TLR5). (B) Calibration plot showing the accuracy of predicted probabilities compared to actual outcomes. (C) DCA validating the clinical utility of the model in decision-making.

10.7717/peerj.20555/supp-13 Supplemental Information 13 ROC analysis of the model using independent validation dataset

10.7717/peerj.20555/supp-14 Supplemental Information 14 qPCR raw data

10.7717/peerj.20555/supp-15 Supplemental Information 15 MIQE checklist

10.7717/peerj.20555/supp-16 Supplemental Information 16 Code

## Additional Information and Declarations

### Data Availability

The following information was supplied regarding data availability: The code and raw data are available in the Supplemental Files, at GEO (GSE66099, GSE13904, GSE26378, and GSE26440), and GitHub (https://github.com/gxmuhjm/exosome_sepsis.git). Huang, J. (2025). codes and raw data in the manuscript. Zenodo. https://doi.org/10.5281/zenodo.17626895.
