# Supplementary material for: Identification and analysis of exosome-associated signatures in pediatric sepsis by integrated bioinformatics analysis and machine learning
Source: PeerJ. 2026 Jan 8;14:e20555. doi: 10.7717/peerj.20555 (PMC12790779; doi:10.7717/peerj.20555)
Supplement: Supplemental Information 1 [file peerj-14-20555-s001.docx]

**Supplementary Table 1**

| Dataset | Platform | Sample size | Sample species | Sample organism |
| --- | --- | --- | --- | --- |
| GSE13904 | GPL570 | 18 healthy and 185 sepsis samples | Homo sapiens | Whole blood |
| GSE26378 | GPL570 | 21 healthy and 82 sepsis samples | Homo sapiens | Whole blood |
| GSE26440 | GPL570 | 32 healthy and 98 sepsis samples | Homo sapiens | Whole blood |
| GSE66099 | GPL570 | 47 healthy and 229 sepsis samples | Homo sapiens | Whole blood |
