# Supplementary material for: Identification and analysis of exosome-associated signatures in pediatric sepsis by integrated bioinformatics analysis and machine learning
Source: PeerJ. 2026 Jan 8;14:e20555. doi: 10.7717/peerj.20555 (PMC12790779; doi:10.7717/peerj.20555)
Supplement: Supplemental Information 2 [file peerj-14-20555-s002.docx]

Supplementary Table 2

| Gene | Primer direction | Sequence |
| --- | --- | --- |
| CD177 | Forward | ATGAGCGCGGTATTACTGCTG |
|  | Reverse | GGTCGGACACCTTCCACAC |
| GYG1 | Forward | TGACACTAACCACAAACGATGC |
|  | Reverse | TAGATGAGCAGAATCGCCACT |
| IRAK3 | Forward | CTGCGGGATCTCCTTAGAGAA |
|  | Reverse | GCAGAGAAATTCCGAGGGCA |
| MCEMP1 | Forward | CCATGCAAAGGGTGGTCATTC |
|  | Reverse | GCTTGTACGGAGTTTGAGACATT |
| TLR5 | Forward | CCGGGTTTGGCTTCCATAACA |
|  | Reverse | TGTGAAAGATCCAGGTGTCTCA |
| β-Actin | Forward | GTCATTCCAAATATGAGATGCGT |
|  | Reverse | GCTATCACCTCCCCTGTGTG |
