# Supplementary material for: Identification and analysis of exosome-associated signatures in pediatric sepsis by integrated bioinformatics analysis and machine learning
Source: PeerJ. 2026 Jan 8;14:e20555. doi: 10.7717/peerj.20555 (PMC12790779; doi:10.7717/peerj.20555)
Supplement: Supplemental Information 6 — (A) The SOFA score for adults. (B) The qSOFA score for adult rapid screening. (C) The pSOFA score for pediatrics. References [file peerj-14-20555-s006.docx]

Supplementary Table 6A The SOFA score for adults.

| **Organ system** | **Variable** | **Score = 0** | **Score = 1** | **Score = 2** | **Score = 3** | **Score = 4** |
| --- | --- | --- | --- | --- | --- | --- |
| Respiration | PaO₂/FiO₂ (mmHg) | >400 | ≤400 | ≤300 | ≤200 (with respiratory support) | ≤100 (with respiratory support) |
| Coagulation | Platelets (×10⁹/L) | ≥150 | <150 | <100 | <50 | <20 |
| Liver | Bilirubin (mg/dL) | <1.2 | 1.2–1.9 | 2.0–5.9 | 6.0–11.9 | >12.0 |
| Cardiovascular | Hypotension | None | MAP <70 mmHg | Dopamine ≤5 or dobutamine (any dose) | Dopamine >5 or epinephrine ≤0.1 or norepinephrine ≤0.1 | Dopamine >15 or epinephrine >0.1 or norepinephrine >0.1 |
| CNS (Glasgow Coma Scale) | GCS | 15 | 13–14 | 10–12 | 6–9 | <6 |
| Renal | Creatinine (mg/dL) or urine output | <1.2 | 1.2–1.9 | 2.0–3.4 | 3.5–4.9 or <500 mL/day | >5.0 or <200 mL/day |

Supplementary Table 6B The qSOFA score for adult rapid screening.

| **Variable** | **Criteria** | **Score** |
| --- | --- | --- |
| Respiratory rate | ≥22 breaths/min | 1 |
| Systolic blood pressure | ≤100 mmHg | 1 |
| Altered mentation | GCS <15 | 1 |

Supplementary Table 6C The pSOFA score for pediatrics.

| **Organ System** | **0** | **1** | **2** | **3** | **4** |
| --- | --- | --- | --- | --- | --- |
| **Respiratory (PaO₂/FiO₂, with mechanical ventilation as required)** | ≥400 | <400 | <300 | <200 | <100 |
| **Coagulation (Platelets, ×10⁹/L)** | ≥150 | <150 | <100 | <50 | <20 |
| **Liver (Total bilirubin, mg/dL)** | <1.2 | 1.2–1.9 | 2.0–5.9 | 6.0–11.9 | >12.0 |
| **Cardiovascular (Mean arterial pressure/vasopressors, adjusted for age)** | No hypotension | MAP < normal (age-adjusted) | Dopamine ≤5 or dobutamine (any dose) | Dopamine >5 or epinephrine ≤0.1 or norepinephrine ≤0.1 | Dopamine >15 or epinephrine >0.1 or norepinephrine >0.1 |
| **Central Nervous System (GCS score)** | 15 | 13–14 | 10–12 | 6–9 | <6 |
| **Renal (Creatinine, mg/dL, age-adjusted)** | Normal (age-specific) | 1.5 × baseline | 2.0 × baseline | 3.0 × baseline | 4.0 × baseline or dialysis |
