# Supplementary figures and images for: Identification and analysis of exosome-associated signatures in pediatric sepsis by integrated bioinformatics analysis and machine learning
Source: PeerJ. 2026 Jan 8;14:e20555. doi: 10.7717/peerj.20555 (PMC12790779; doi:10.7717/peerj.20555)

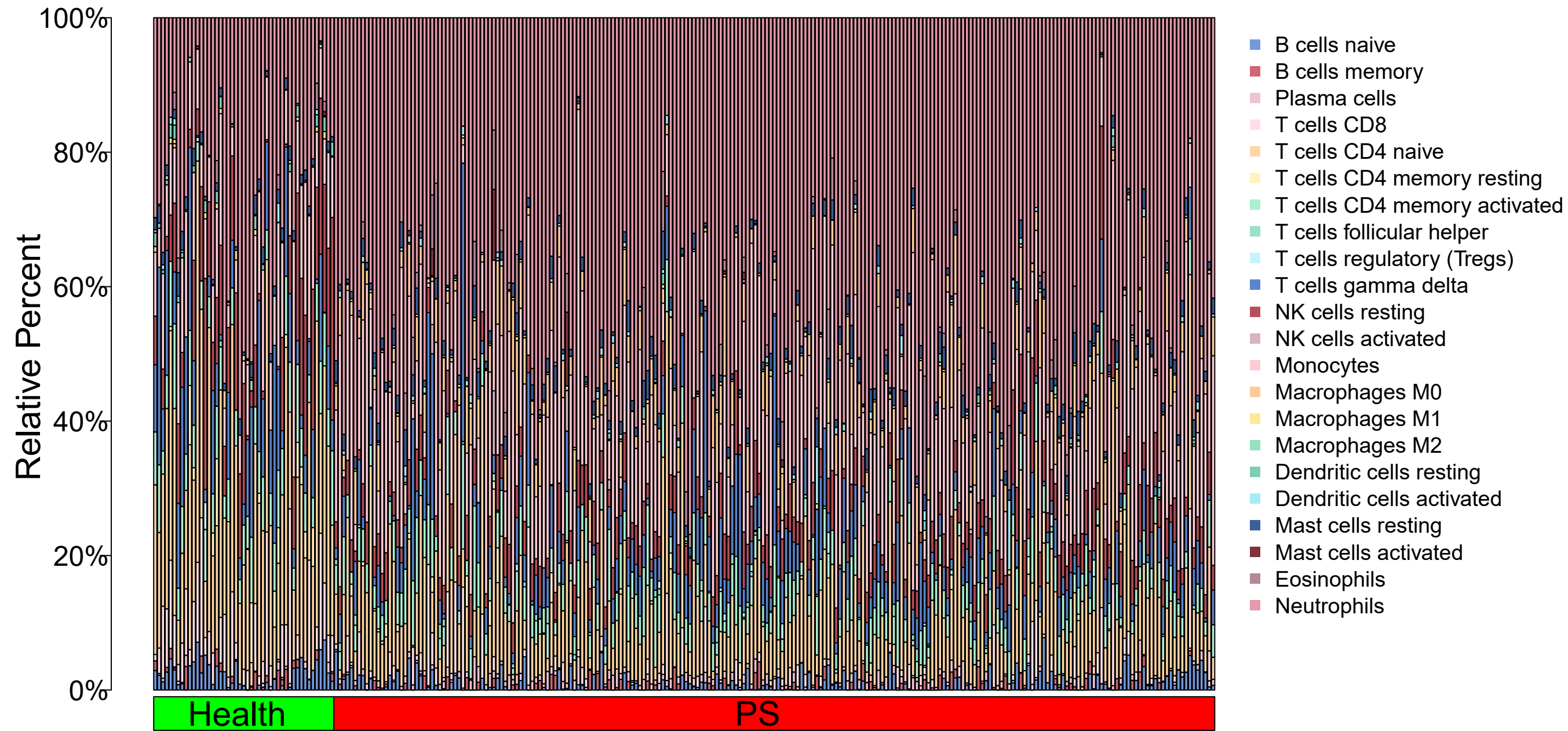

Supplement: Supplemental Information 7 [file peerj-14-20555-s007.pdf]

A

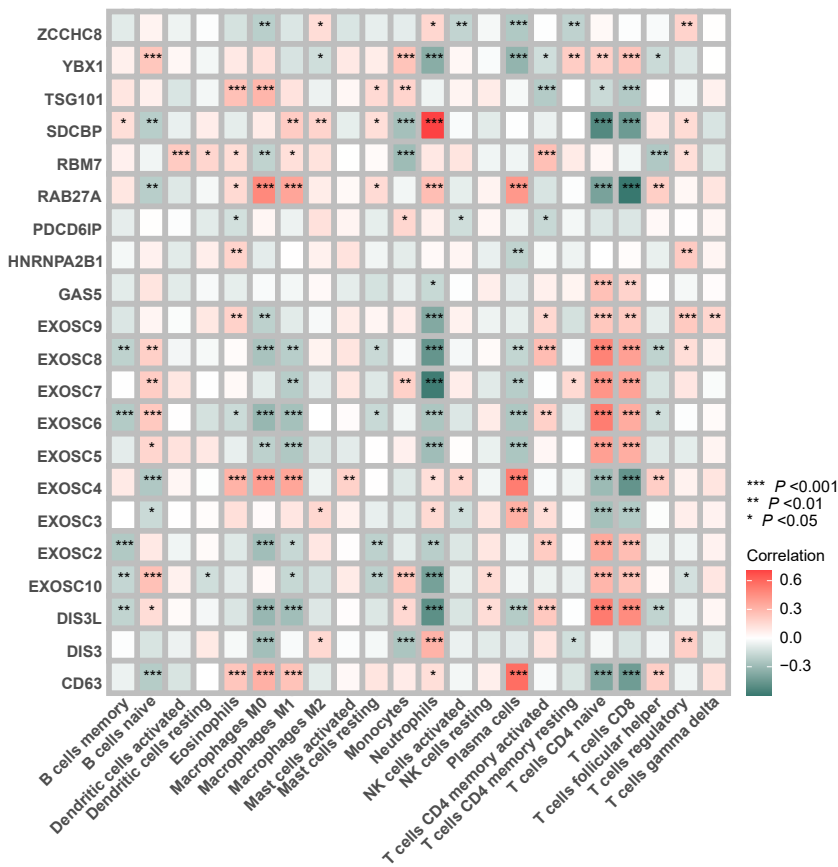

B

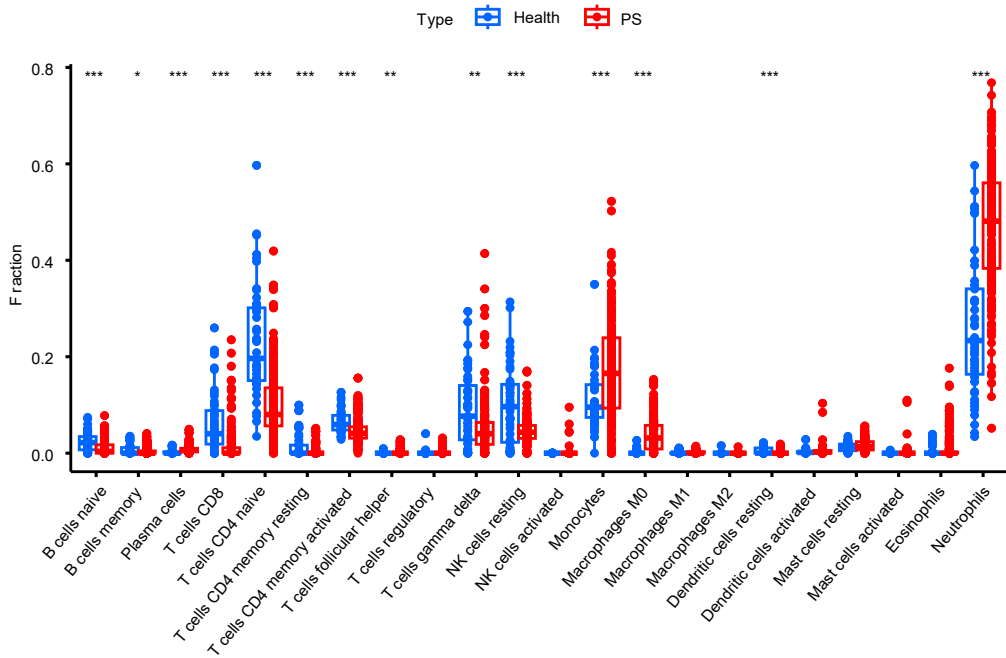

Supplement: Supplemental Information 8 — (A) Correlation analysis demonstrating the interrelationships among 22 differentially expressed ERGs. (B) Violin plot indicating the differential abundance of infiltrating immune cells between PS and healthy samples. [file peerj-14-20555-s008.pdf]

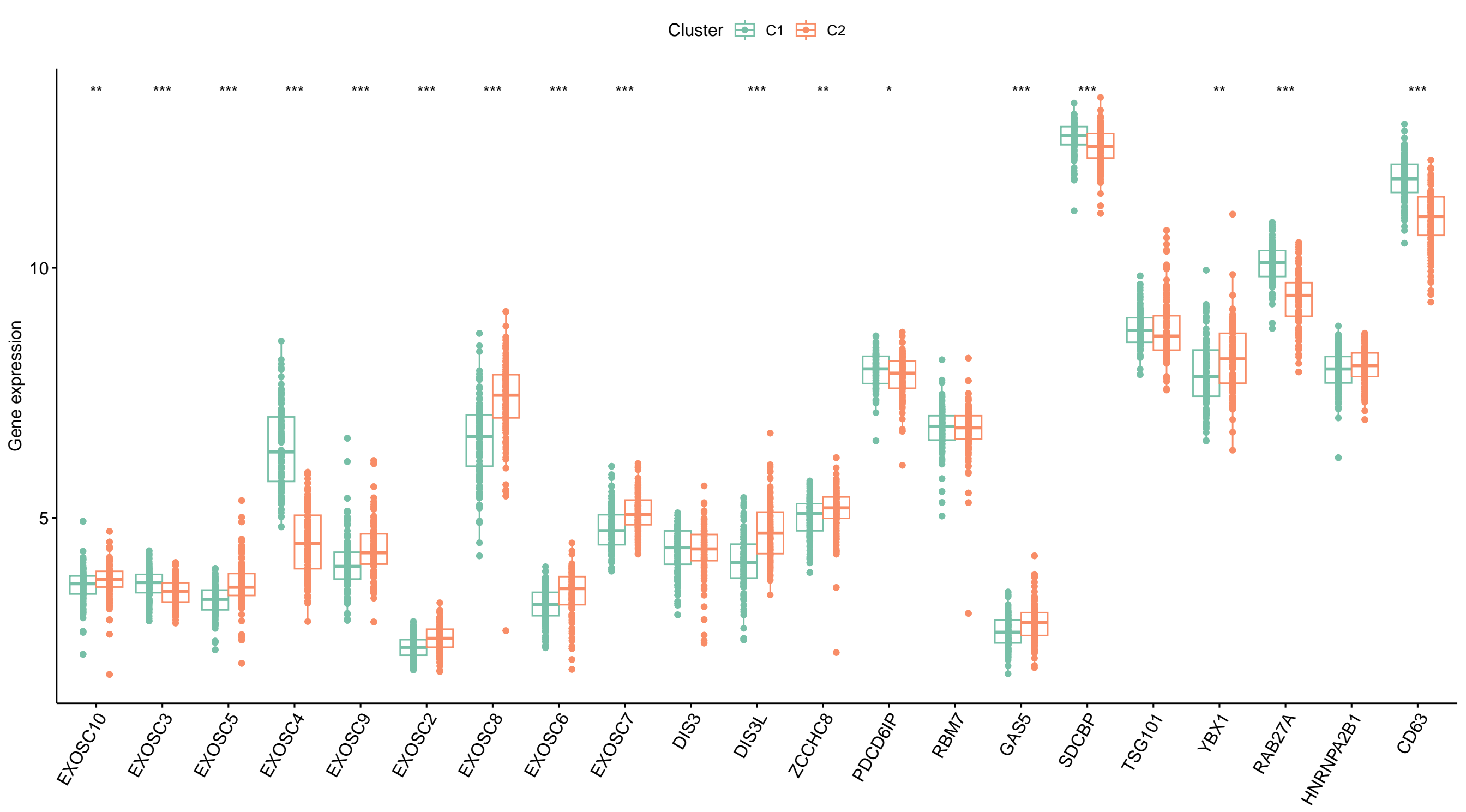

Supplement: Supplemental Information 9 [file peerj-14-20555-s009.pdf]

**A**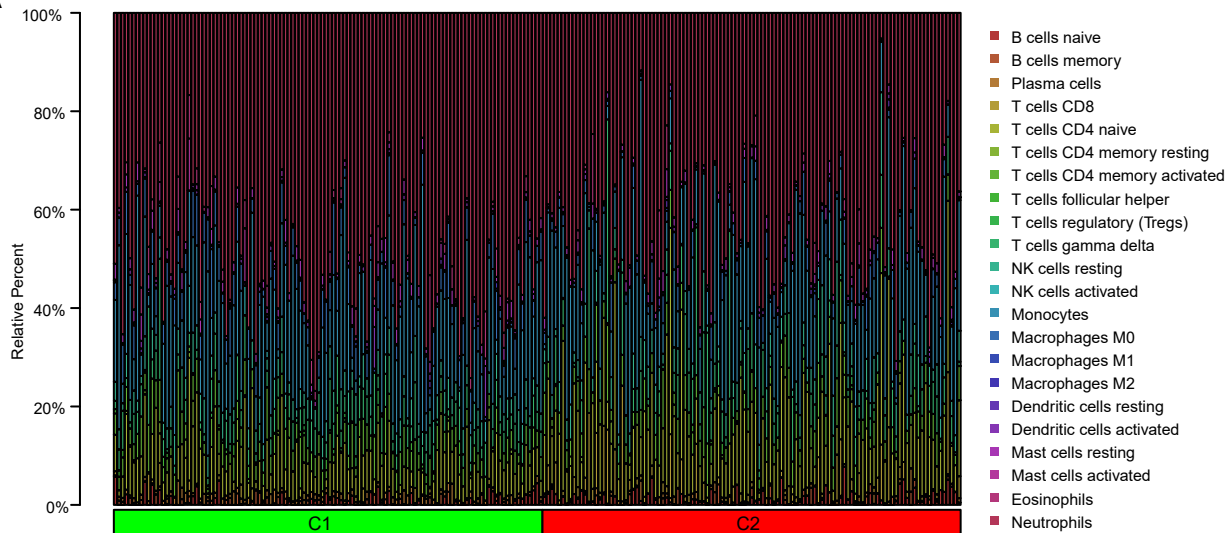**B**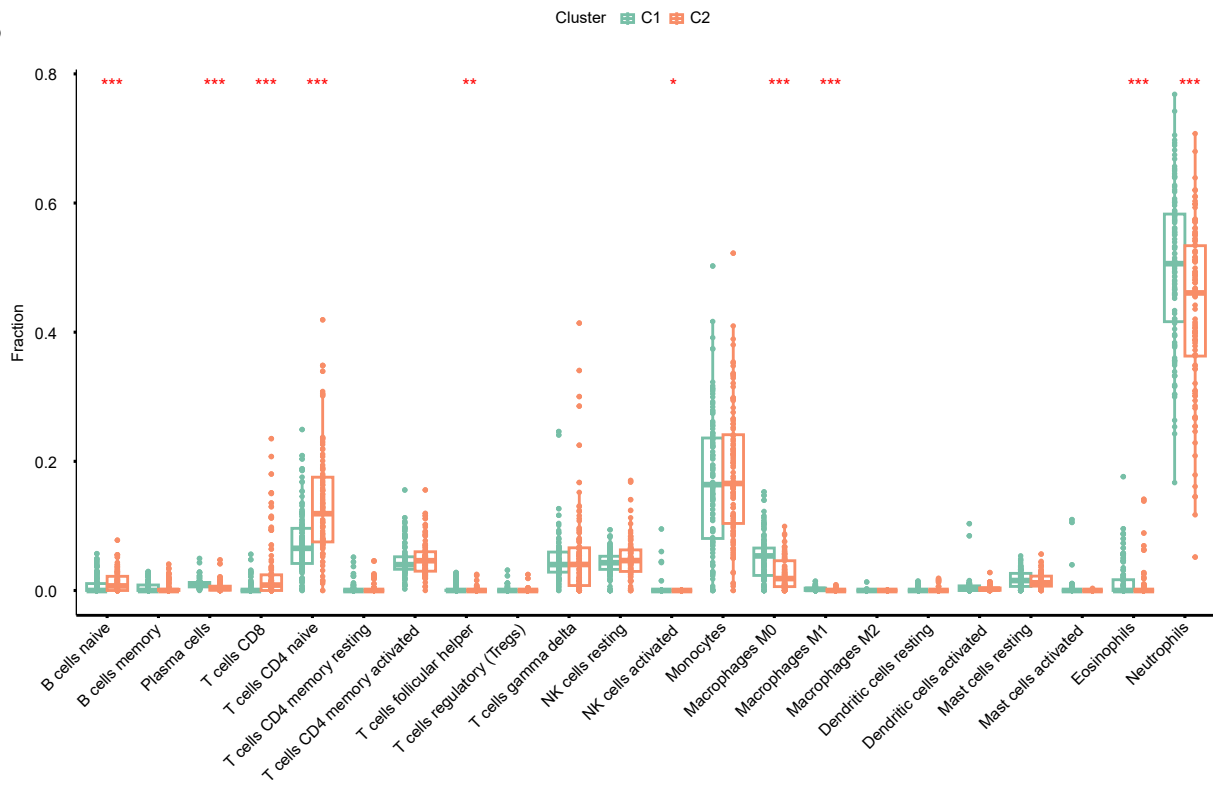

Supplement: Supplemental Information 10 — (A) Proportion of 22 immune cell types infiltrating the clusters. (B) Violin plot demonstrating the differential immune cell abundance between C1 and C2 clusters. [file peerj-14-20555-s010.pdf]

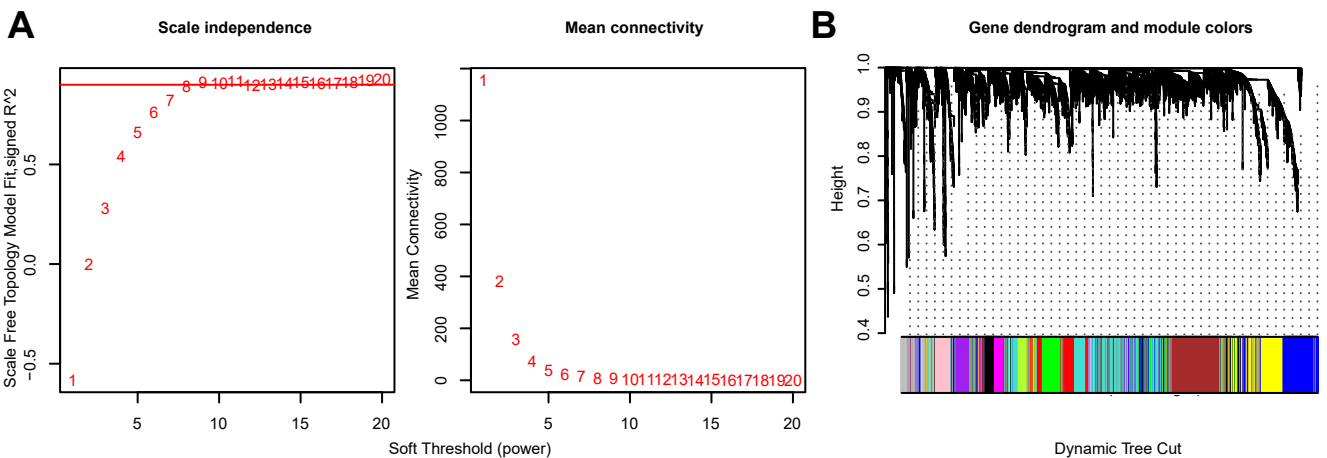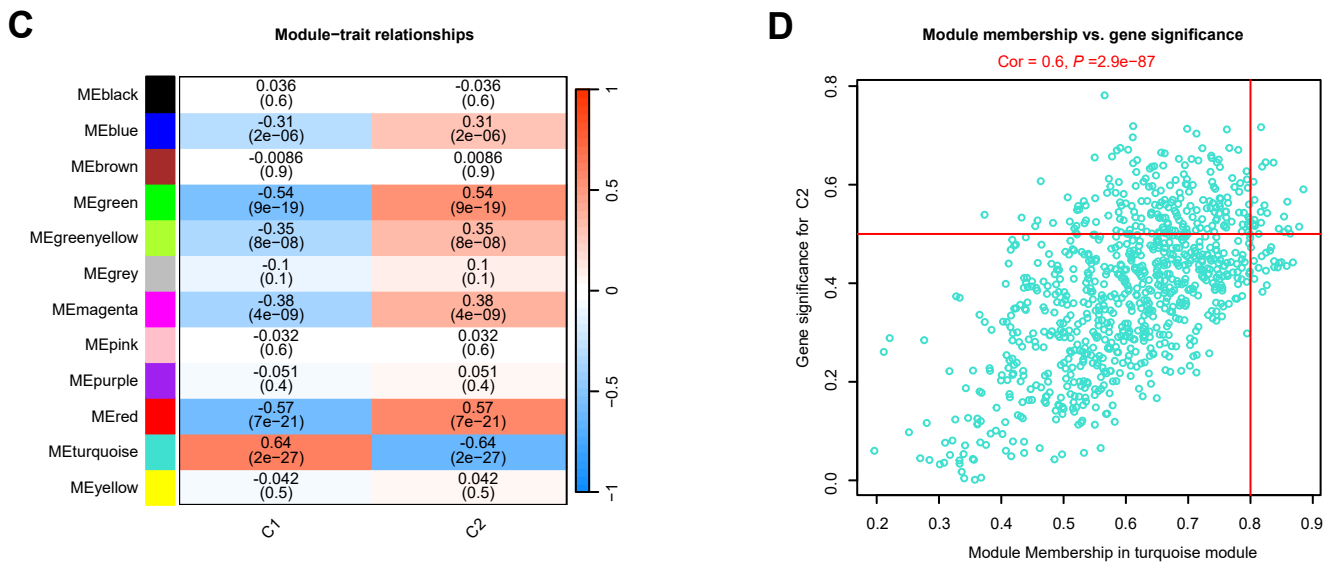

Supplement: Supplemental Information 11 — (A) Soft-thresholding procedure used for network construction. (B) Hierarchical clustering dendrogram of genes. (C) Correlation heatmap showing the correlation between ERGs modules and cluster features. (D) Association of turquoise module genes with exosome-related processes. [file peerj-14-20555-s011.pdf]

**A**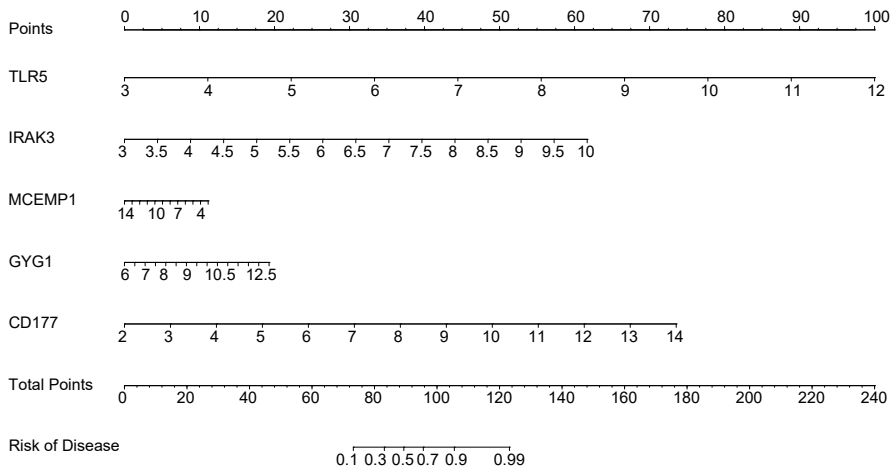**B**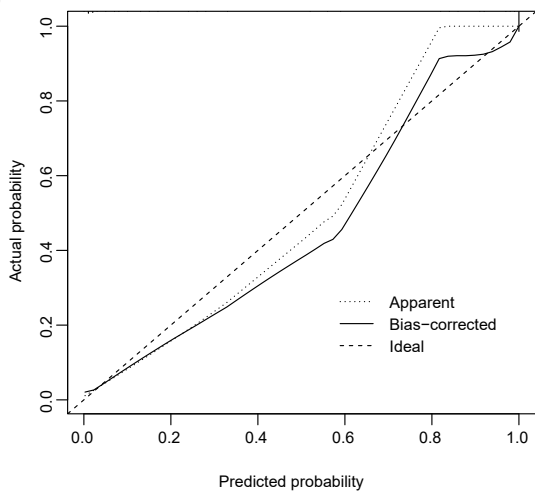**C**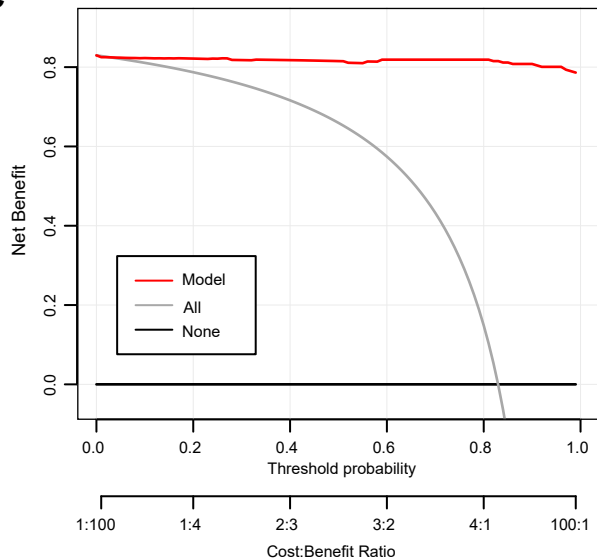

Supplement: Supplemental Information 12 — (A) Nomogram constructed using the 5 hub genes (CD177, GYG1, IRAK3, MCEMP1, TLR5). (B) Calibration plot showing the accuracy of predicted probabilities compared to actual outcomes. (C) DCA validating the clinical utility of the model in decision-making. [file peerj-14-20555-s012.pdf]

**A****GSE13904**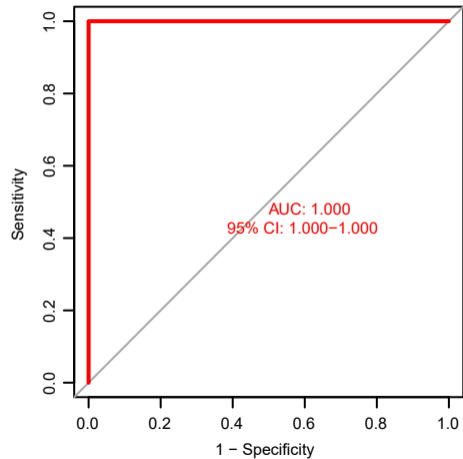**B****GSE26378**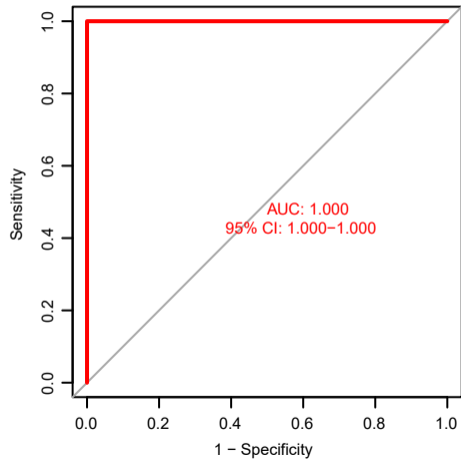**C****GSE26440**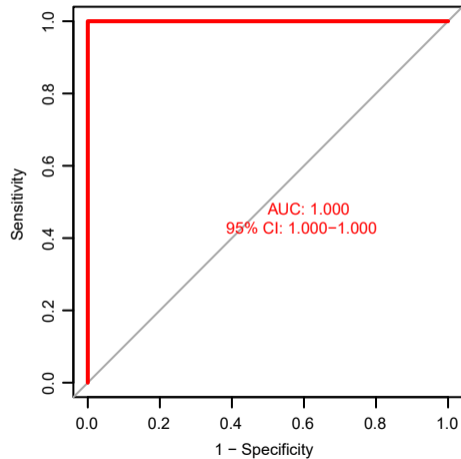

Supplement: Supplemental Information 13 [file peerj-14-20555-s013.pdf]
